# Barriers and opportunities for improving management of snakebites: Perspectives of healthcare workers in Northern Uganda

**DOI:** 10.1371/journal.pone.0291032

**Published:** 2023-09-25

**Authors:** Solomon T. Wafula, Lydia N. Namakula, Lesley R. Ninsiima, Noah Kiwanuka Ssekamatte, Abel W. Walekhwa, Innocent B. Mugume, David Musoke

**Affiliations:** 1 Department of Disease Control and Environmental Health, College of Health Sciences, Makerere University, Kampala, Uganda; 2 Department of Infectious Disease Epidemiology, Bernhard Nocht Institute for Tropical Medicine (BNITM), Hamburg, Germany; 3 Diseases Dynamics Unit, Department of Veterinary Medicine, University of Cambridge, Cambridge, United Kingdom; 4 Ministry of Health, Kampala, Uganda; BOKU: Universitat fur Bodenkultur Wien, AUSTRIA

## Abstract

**Background:**

Snakebites are a neglected public health problem that pose a significant burden on affected individuals and communities in many sub-Saharan African countries, including Uganda. However, the barriers and facilitators to snakebite management within healthcare settings are not as well understood and well-documented. The aim of this study was to explore the experiences and perspectives of healthcare workers involved in handling snakebite incidents at individual and health system levels in Arua and Gulu districts in Northern Uganda. We sought to understand how healthcare workers manage snakebite cases, what challenges they encounter, and what opportunities they perceive for improvement.

**Methods:**

We conducted a qualitative study using in-depth interviews with 18 healthcare workers from different cadres, seniority levels, and facility types. We used iterative thematic analysis to explore the management procedures, challenges, and opportunities for snakebite management. Using thematic analysis, we identified the overarching themes and subthemes related to snakebite management and associated barriers and opportunities.

**Results:**

The main barriers to snakebite management identified by healthcare workers were inadequate knowledge and skills; limited availability of antivenom; lack of protocols for snakebite management; delayed treatment-seeking for patients; and poor referral systems. The main opportunities for improvement were regular in-service training; increasing public education and awareness about snakebite prevention and management; and increased funding and research.

**Conclusion:**

This study highlights the need for interventions to address the identified barriers while leveraging the existing opportunities to enhance snakebite management in Uganda. Specifically, we recommend the provision of regular training and support to healthcare workers, developing clinical guidelines, and improving the availability of antivenoms.

## Introduction

Snakebite envenomation (SBE) kills more people than any other neglected tropical disease, yet it remains underfunded and under researched globally [1]. About five million people worldwide suffer from snakebites every year, resulting in over 100,000 deaths and serious disabilities [2]. The majority of snakebite victims live in low- and middle-income countries (LMICs), where access to health care and antivenom is limited and variable. In Africa alone, 435,000 to 580,000 people are affected by snakebites annually [2, 3]. SBE can cause severe multi-organ or multi-system damage, such as hemorrhage, paralysis, tissue necrosis, muscle breakdown, cardiotoxicity, acute kidney injury, hypovolemic shock, and death [4]. Therefore, snakebite is a medical emergency that requires prompt and accurate diagnosis and treatment [5]. However, the availability and quality of antivenom and other snakebite therapeutics are inadequate and variable across regions [6, 7] Moreover, healthcare workers often lack formal training and knowledge on how to manage snakebite cases effectively, including providing first aid, identifying snake species, administering antivenom, and monitoring patients [8, 9].

In Uganda, snakebite affects 101 out of every 100,0000 people annually, with the Northern region being one of the high-incidence areas due to its tropical location and diverse snake fauna [10, 11]. However, the disease burden and the social and economic impacts of snakebite are difficult to estimate because of poor data collection, limited access to health care, and low awareness among affected communities [2]. Moreover, Uganda has a variety of snake species with different venom profiles [11] which poses a challenge for the clinical diagnosis and treatment. Different snake species require different types of antivenom and treatment protocols, which are often unavailable or inadequate in most health facilities in Uganda [12].

There is an urgent need for drug discovery programs to expand the snakebite drug portfolio and to overcome the challenges of developing single-drug or combination drug therapies for different types of envenomation [6]. Moreover, improving the health system capacity and performance for snakebite care is essential for achieving universal health coverage (UHC) by 2030, as it involves the same components as providing quality health care for all people [13]. In Uganda, there is a lack of studies on the burden of snakebites and the health facility capacity to manage this public health challenge. Moreover, most facilities do not have antivenom and drugs for supportive treatment, which compromises the quality of snakebite care. While other countries have identified factors such as inadequate regulatory framework, limited funding, and high hospital costs as barriers to effective snakebite management, no such studies have been done in Uganda [8, 14]. Therefore, this study aimed to explore the perspectives of healthcare workers on the management of snakebites, including the challenges and opportunities for improving the situation.

## Methods

### Study setting

**Fig 1:**
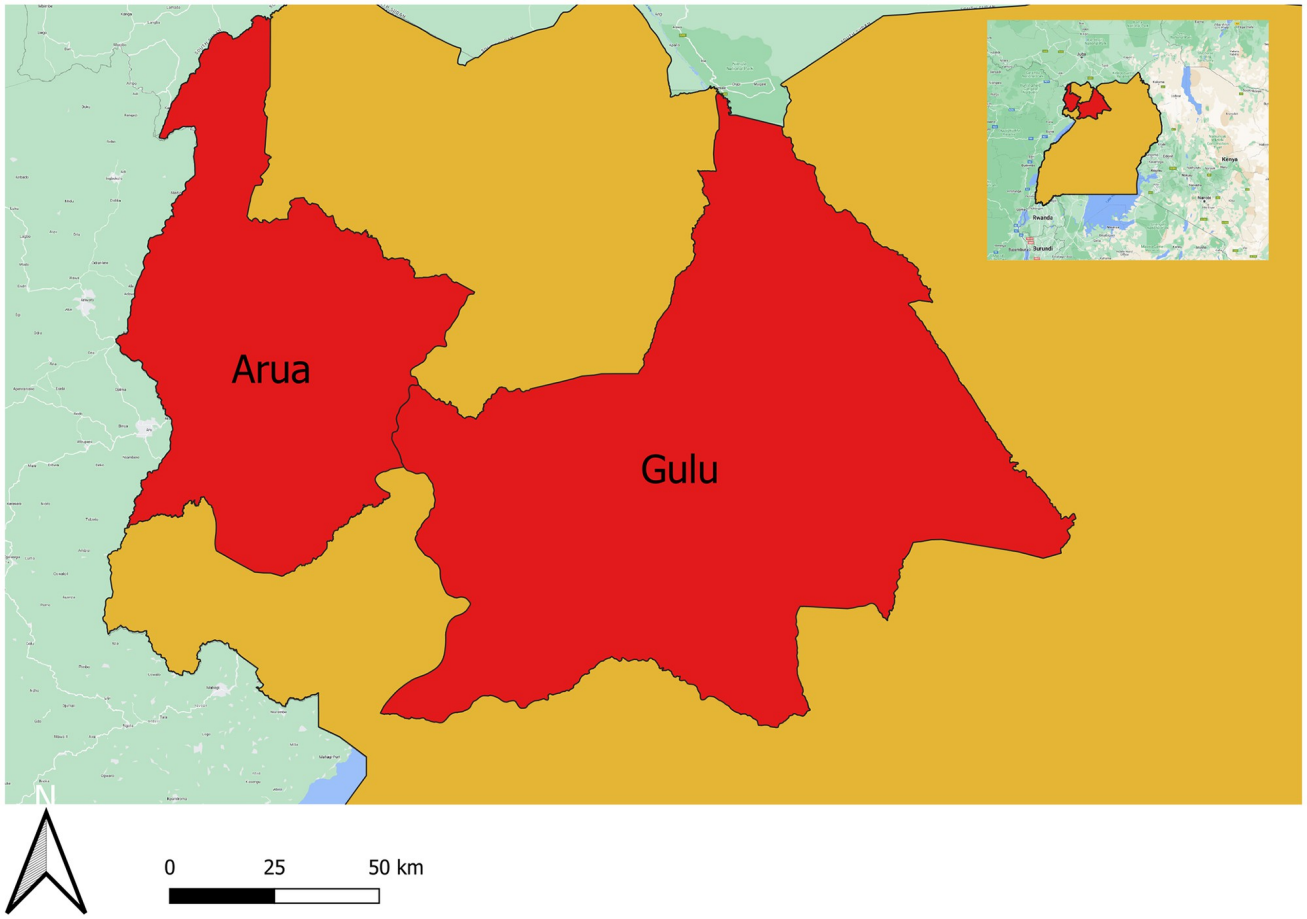
Map of Uganda showing Arua and Gulu districts.

The study was conducted in Arua and Gulu districts of Northern Uganda, which are endemic for snakebite envenomation (Fig 1). The region has a tropical climate with a single rainy season from March to October, in contrast to the rest of Uganda which has two rainy seasons [15]. The temperature ranges from 15°C in July to 30°C in February, with an average of 25°C [16]. These conditions favor snake habitats, especially when there is plenty of vegetation and food during the rain. Some of the snakes commonly found in the region are the black mamba, the puff adder, the cobra, and the python [17]. These districts are mostly rural, with many bushes providing good conditions for snakebites [18]. Gulu district has a population of 443,733 people and 65 health facilities (33 public, 15 private and 17 private-not-for-profit (PNFP)), while Arua district has a population of 750,000 people and 58 HCFs (36 public, 17 private, and 5 PNFPs). The regional referral hospitals have different categories of healthcare workers who handle snakebite cases at both outpatient and inpatient departments. The healthcare workers receive training from different training, and are registered and licensed by different professional bodies under the Ministry of Health.

### Participants

We recruited 18 healthcare workers from four HCFs in Arua and Gulu districts using purposive sampling in May 2022. The HCFs included two public regional referral hospitals (Arua and Gulu) and two private-not-for-profit hospitals (Pioneer and Lacor). The healthcare workers included 7 males and 11 females. They had different professional backgrounds, such as physicians, nurses, pharmacists, laboratory technicians, and clinical officers. They also had different levels of experience in managing snakebite cases, ranging from 1 to 15 years. We selected the healthcare workers based on their involvement in triage, diagnosis, treatment, or laboratory work related to snakebite management. We obtained administrative clearance from the director or in-charge of each facility and informed consent from each participant before conducting the interviews.

### Data collection procedures

We used an in-depth interview (IDI) guide to collect data from the healthcare workers. The IDI guide consisted of open-ended questions that explored the healthcare workers’ challenges and opportunities for improving snakebite management. The questions were developed based on the input of neglected tropical diseases (NTD) clinical and research experts and a review of the existing literature on snakebite management [19–21]. The IDI guide was pilot-tested with two healthcare workers from a different facility and revised accordingly. The IDI guide had good face validity and content validity as assessed by the research team. We then conducted semi-structured interviews with the healthcare workers using the IDI guides in English language. The interviews were conducted face to face by three male research assistants who had training in qualitative research methods and held at least a bachelor’s degree in medical sciences. The interviews took place at a convenient time and location for each participant, either at their workplace or at a nearby office. No external individuals were present during the interviews. The interviews lasted between 25 to 50 minutes and were audio-recorded with the permission of the participants. We also took field notes during and after each interview to capture our observations and reflections. We stopped data collection when we reached theoretical saturation [22], meaning that no new information or themes emerged from the interviews.

### Research team and reflexivity

#### Personal characteristics

The interviews were conducted by three male research assistants with prior experience in qualitative research interviewing. The investigation team consisted of a physician with vast snakebite clinical experience, as well as a clinical research expert with a Master of epidemiology.

#### Connection with participants

There was no history between interviewers and any of the participants. All participants were first informed by their unit leads about the invitation to participate and schedule an interview date. At the start of the interview, the interviewers made a personal introduction to each participant and gave a brief overview of their position within the study.

### Data analysis

We transcribed the audio recordings verbatim and checked them for accuracy. We then imported the transcripts into Atlas ti software for coding and analysis. Data were analyzed through thematic analysis by two researchers. Using Atlas ti, these researchers independently coded the transcripts, marking sections of text that contained important concepts and giving them descriptive labels. They then reviewed their codes and reached a consensus on a final coding scheme by making iterative adjustments. The full coding scheme is available (S1 File). From the codes, they identified themes and subthemes that reflected the patterns and topics in the data. They also explored how the themes and subthemes were related to each other and to the research questions. We ensured the trustworthiness of the analysis through peer debriefing, and reflexivity.

### Ethical considerations

We followed ethical guidelines and obtained approval from two relevant institutions: Makerere University School of Public Health Higher Degrees, Research and Ethics Committee (HDREC Ref No: SPH-2021-210) and Uganda National Council for Science and Technology (UNCST; Ref No: HS2121ES). We respected the autonomy and confidentiality of the participants. We provided them with a participant information sheet in English, which clearly explained the purpose and process of the study. They were given ample time, at least an hour, to carefully review the information before granting their written informed consent. We also answered any questions they had and introduced them to the research assistants who would conduct the interviews. We did not collect any personal identifiers from the participants and we stored and backed up the data securely on passcode-protected drives that only the principal investigator could access.

## Results

### Participant characteristics

Out of 22 healthcare workers invited, 18agreed to participate in this study. These included 10 nurses (55.6%), 5 medical officers or consultants (27.8%), and 3 laboratory personnel (16.7%). The healthcare workers included 7 males (38.9%) and 11 females (61.1%), with a mean age of 35 years (SD = 5.6). They had different levels of experience in managing snakebite cases, ranging from 1 to 15 years (M = 6.7, SD = 4.2). The majority of participants (11) worked at Arua Regional Referral Hospital, while 2 were based at Gulu Regional Referral Hospital, 1 at Rheema Hospital, and 4 at St. Mary’s Hospital Lacor (Table 1).

**Table 1:** Participant recruitment per health facility.

Characteristics.	Number (*N* = 18)	Percent (%)
**Sex of the participants**		
Male	07	38.9
Female	11	61.1
**Cadre**		
Nurses	10	55.6
Medical officers or consultants	05	27.8
Laboratory personnel	03	16.7
**District**		
Arua	12	66.7
Gulu	06	33.3
**Health facility**		
Arua Regional Referral Hospital	11	61.1
Gulu Regional Referral Hospital	2	11.1
Rheema hospital	1	5.6
St. Mary’s hospital Lacor	4	22.2
Age in years [Mean (SD)]	18	35.6 (9.5)
**Years of experience** [Mean (SD)]	18	6.7 (4.2)

### Emerging themes

The main emerging themes in the management of snakebite cases included knowledge about management procedures, including the use of clinical guidelines and supportive treatments such as local first aid; the importance of healthcare workers’ knowledge on signs, symptoms, and species identification for effective management. Challenges identified include the lack of up-to-date robust clinical guidelines, delayed health-seeking behavior, limited knowledge and skills among healthcare workers, inadequate availability and high cost of antivenoms, and poor referral systems. Opportunities for improvement involve increasing awareness and education among healthcare workers and the general public, conducting research for better management practices and alternative treatments, and securing funding for adequate resources.

### Snakebite management procedures and health workers’ knowledge

#### Management procedure

The participants expressed knowledge about snake management at health facility level, as well as acknowledged that snake bites are dangerous and if not timely treated, can result in permanent disability, disfigurement, or even death. Fortunately, they self-reported/appraised themselves on the need to use clinical guidelines in the management of snake bites. For example, the participants noted that management of cases was based on the snakebite management guidelines and any available health facility-specific protocols. The participants showed knowledge on the supportive treatment procedures including local first aid and history taking from the cases that happen at the health facilities which is usually done when a patient is rushed there.

“…*When you are rushed to the hospital before the anti-venom is given*, *they first give you IM or IV of hydrocortisone to neutralize the poison it has left in your body*. *After neutralizing with hydrocortisone*, *you are given also IV (antihistamines) to give you strength*. *Then you are given anti-snake venom and you are also subjected to a PPF [procaine penicillin fortified] treatment of 5 days*."

(Nurse -Screening and linkage, IDI–1)

Participants emphasized that history taking and the local first aid provided at home were important for determining the type and severity of the snake bite. They emphasized these procedures give good guidance to guide the kind of supportive treatment to provide to the patients.

“*If the client has given you good history of the snakebite and the type of the snake which has bitten him or her*, *then that is what we normally rely on that to give the treatment*. *Because there is not normally much investigation [such as] those laboratory investigations to determine whether this one (snake involved) is poisonous*.”

(Nursing officer, IDI–6)

The study found that snake envenomation treatment involved first aid and adjuvant interventions. Some of the health facilities had a displayed protocol for managing patients, which most healthcare workers referred to. They also mentioned the Uganda Clinical guidelines 2016 and 2020 as reference materials for all health-related conditions and procedures. One nurse remarked

*"[When handling these cases]*, *we always refer ourselves to Uganda clinical guidelines*, *which provides brief information about [management of] those bites and stings*. *All the healthcare workers are given clinical guidelines of 2007*, *not the new updated one of 2020*, *because very few of them got it*.*"*

(Nurse, IDI–13)

#### Snakebite diagnosis based on signs and symptoms

The study explored the knowledge and practices of healthcare workers regarding snakebite management. One key aspect was the process of identifying the type of snake that caused the bite. The participants admitted that patients often cannot provide accurate information about the snake’s color or species. Therefore, healthcare workers diagnose snakebites based on signs and symptoms. For example, for neurotoxic snakebites, they look for sweating, emasculation, and drooling of saliva. For cytotoxic snakebites, they look for swelling. For hemotoxic snakebites, they look for active bleeding. This approach helps healthcare workers to make a diagnosis even without detailed information about the snake. One participant remarked as follows

*" We first try to ascertain which type of snake*? *Did they see the color or not*? *But sometimes*, *for them*, *they fail to see the type or the color of the snake*. *So*, *we always diagnose [suspect diagnosis] by basing on signs and symptoms*. *As for neurotoxic snakebites*, *you look for signs; sweating*, *and then drooling of saliva*. *That one we can easily diagnose*. *Then those ones with cytotoxic*, *we see the swelling*. *Then for hemotoxic*, *we always see active bleeding*.***"***

(Nurse, IDI–8)

### Challenges faced in the management of snakebite cases

#### Lack of up-to-date robust clinical guidelines

Although some healthcare workers had self-reported their knowledge and utilization of Uganda clinical guidelines, they highlighted several barriers. For instance, except for Lacor Hospital which had comprehensive protocols for snakebite management, healthcare workers from other facilities highlighted a lack of robust clinical guidelines on snakebite management. They pointed out gaps in the national guidelines for snakebite cases. For example, the supportive treatment recommended in the guidelines was considered weak, and the guidelines were noted to be insufficient in detail.

One participant remarked as follows

“*However*, *within the clinical guidelines*, *there exists a notable weakness*. *it suggests administering chlorphenamine*, *a relatively weak antihistamine*, *in tablet form*. *This is a concern when dealing with severe cases of envenomation*, *where patients may show clear signs of venomous effects*. *Given the circumstances*, *the efficacy of chlorphenamine is questionable and not deemed as a viable option*.*”*

(Clinical officer, IDI–18)

#### Delayed health-seeking behavior for patients

Healthcare workers mentioned the challenge of patients arriving late for medical attention at the health facility. This delay in seeking healthcare services for snakebite cases was seen as a major obstacle in managing such cases effectively. The healthcare workers said that this delay often led to adverse outcomes, such as poor health and sometimes death.

“*One or two (patients) that pass away [in a year] […* .*] in most cases*, *it is because they delayed to reach the facility*. *These individuals arrive late*, *so by the time we are attempting to admit them for medical care [Hospitalize]*, *they are already in dire circumstances*. *For most of the people that come early*, *they are helped*, *given care*, *and they* recover. Of course, we’ve had delays [many instances where patients are presented to hospital late] many hours after the snakebite. At times, the patient checks in the following day when the whole body is paralyzed. At this point, I really can’t do much [to save the patient]. It is one of the biggest challenges”.

(Medical officer, IDI–16)

#### Limited knowledge and skills

Many healthcare workers in the study expressed a lack of adequate knowledge and skills in managing snakebite cases. They reported challenges in areas such as identifying snake species and applying appropriate treatment protocols. This lack of knowledge and skills raises concerns about the effectiveness of their interventions in snakebite management.

*“I have seen that many of my colleagues in health care*, *they don’t have enough knowledge about how to deal with snakebite cases*. *It doesn’t matter if they are nurses or doctors or specialists*, *they all struggle with snakebite management*. *Even myself*, *I have to admit that I’m not very confident in my skills*. *We have a big gap in our knowledge in this area*, *and it’s a very serious concern for us*.*”*

(Medical officer, IDI–15)

At administrative level, participants highlighted that health facilities prioritize capacity building for other medical conditions over snakebite case management even in endemic districts.

*“People are eager to learn more about snakebites*, *but they are left behind*. *The government and other partners don’t care about us*, *they only care about their own agendas*. *They ignore the need for proper training on snakebites*. *This makes us feel frustrated and helpless*, *because we can’t improve our knowledge and skills*.*”*

(Medical officer, IDI–15)

#### Lack of antivenoms and high cost of treatment

Lack of resources, especially adequate supplies of anti-venom, was a major obstacle in managing snakebites effectively, as healthcare workers reported. This shortage of anti-venom in health facilities, along with the high cost of treatment, posed significant challenges, especially for victims who could not afford the necessary care

*“Sometimes they bring just about 10 doses of anti-venom for the whole three months [for entire hospital]*. *Can you imagine*? *It’s barely enough*! *And if it’s not available when a patient needs it*, *we have no choice but to ask them to buy it themselves*. *But you know*, *many of them cannot afford it*. *So instead*, *we have to make work with what we have [all alternative treatments available]*. *We give them hydrocortisone and antibiotics*, *but deep down*, *we know it’s not enough*. *We can only pray and hope for the best because there’s really nothing more we can do*.*”*

(Senior nursing officer, IDI–5)

#### Poor referral systems

Healthcare workers mentioned the challenges caused by poor referral systems and poor communication between health facilities, leading to difficulties in providing timely and appropriate treatment to snakebite patients. Participants stressed that the current ways of patient referral were unreliable, worsening the problem.

“*We feel very bad when we see our patients suffering because of the problems in the referral system*. *Some patients are kept by other facilities for too long*, *even when they have serious conditions like cytotoxicity*. *When they come to us*, *their situation is very bad*. *Others need to go to higher-level facilities where they can get the right treatment*, *like antivenom for snake bites*. *But this process is also slow and can put their lives in danger*. *We wish we could do more to help our patients and give them the best care possible*.”

(Nurse, IDI–8)

### Opportunities for better management of snakebite cases

#### Increasing awareness and education about snakebite management among healthcare workers through training and workshops

The participants emphasized that sharing experiences among themselves would be a valuable learning platform and an opportunity for professional growth. They recognized that snakebite cases can be challenging and complex, and by exchanging their experiences, healthcare workers who have never encountered such cases would benefit from the practical knowledge and insights of their colleagues. Through discussions and interactions, they could learn about different snakebite scenarios, the specific management approaches used, and the lessons learned from previous cases

*“We need to attend those seminars because they help us to update our knowledge*. *The protocols keep changing*, *so we have to learn new things*. *We also get CMEs [Continuous medical education]*, *which are good for our learning and our profession*. *We can also use Google [evidence search] to see how other hospitals are managing snakebite cases*. *It helps us to improve our practices and give better care to our patients”*

(Nurse, IDI–13)

#### Increasing education and awareness about snakebite prevention and management in the general public

Healthcare workers strongly emphasized the importance of community sensitization to enhance the understanding and knowledge of individuals and communities regarding the initial management of snakebite cases before reaching a health facility. They highlighted that by raising awareness and providing education in communities, individuals can learn valuable information and skills to respond quickly to snakebite incidents. This proactive approach empowers community members to administer appropriate first aid measures, which can significantly improve outcomes for snakebite victims

*“Besides the CMEs*, *we should also integrate snakebite management with other healthcare activities when we go out for outreaches*. *For example*, *when we do surveillance for diseases like TB*, *COVID-19*, *or malaria*, *we should also look into snakebite cases*. *This would help us to know the situation in the community or the health center*. *We can check the register and see how many people have come for snakebite treatment in that health center*. *This integrated approach would help us to meet the comprehensive healthcare needs of the people*.*”*

(Consultant physician, IDI–4)

#### Research into better snakebite management practices and alternative treatments for snakebite, to complement or replace antivenom

One of the important opportunities highlighted was the need for research into improving snakebite management practices and finding alternative treatments to complement or replace antivenom. The participants recognized that current management approaches may have limitations and that there is a desire to find more effective solutions. This highlights the importance of ongoing research and innovation in snakebite management. One participant mentions

*“I believe that conducting studies like this will greatly benefit us as medical practitioners*. *Such studies help us stay updated and informed*. *Your study on the management of snakebites is a valuable opportunity for our facility*, *as it allows us to better prepare ourselves for handling snakebite cases*. *This knowledge is crucial for us working in rural areas where snakebites are common*.*”*

(Medical Officer, IDI—14)

#### Funding and budgeting considerations for supplies

Participants highlighted the importance of funding as a key opportunity that could improve the health facility budget and the availability of necessary supplies for snakebite case management. Adequate funding would address the ongoing challenges of limited resources and shortages in essential items such as anti-venom, which are vital for treating snakebite patients. With increased financial support, health facilities would be better equipped to ensure a consistent supply of anti-venom and other medications, as well as invest in training programs for healthcare workers to enhance their skills and knowledge in snakebite management. One healthcare worker remarked as follows

*“Yeah*, *when it comes to snake anti-venom*, *it’s not always readily available*. *We can still obtain it when needed*, *but not immediately [Not readily available]*. *I am aware that I have the authority to prescribe it*, *but there are certain strict regulations surrounding its prescription*. *I believe it’s due to its limited availability*. *If there were an abundant supply*, *say 10 vials without any shortage*, *then I would feel more confident [to easily prescribe it to patients]*. *However*, *I’m concerned that in a worst-case scenario*, *I might not be able to obtain it*. *So*, *proper budgeting is necessary to ensure its availability*.*”*

(Medical Officer, IDI–16)

## Discussion

The study’s findings have significant implications for public health, highlighting challenges and opportunities in snakebite management. These challenges include the lack of robust clinical guidelines, delayed health-seeking behavior, limited knowledge and skills among healthcare workers, and inadequate availability of antivenoms. Addressing these challenges can improve the timely and effective management of snakebite cases, reducing disability and mortality rates. Opportunities such as increasing awareness and education, conducting research on alternative treatments, and securing funding for supplies can enhance snakebite prevention and patient care. Implementing these strategies strengthens public health systems’ ability to mitigate the impact of snakebites and improve health outcomes for affected communities.

The study reveals that the current Uganda clinical guidelines lack comprehensive information on the management, toxicity, and clinical presentation of snakebites, as well as appropriate supportive treatment. One of the key factors for optimal patient outcomes in snakebite management is the availability and adherence to up-to-date guidelines that provide specific and evidence-based recommendations for different scenarios of bites, toxicity and clinical presentation [23]. However, in Uganda, the current clinical guidelines have limited information on these aspects and do not address the appropriate supportive treatment for snakebite patients [24]. Moreover, snakebite is clustered under the bites and stings category and does not have its own separate guidelines [24]. This may result in poor awareness and ineffective treatment among healthcare providers, leading to increased morbidity and mortality for snakebite patients. One of the main challenges for developing comprehensive and updated clinical guidelines for snakebite management is the long time it takes to revise the guidelines and the lack of research in this area, as snakebite is a neglected tropical disease that receives insufficient funding and attention compared to other diseases. Therefore, we urge the Ministry of Health to conduct a detailed review of the existing snakebite guidelines and incorporate the latest evidence and best practices from other regions where snakebite is endemic. This would help to improve the emergency care and long-term outcomes for snakebite patients in Uganda.

We found that many patients presented late for treatment after snakebite, which increased their risk of severe complications and death. The causes were suggested as cost or transport challenges but also use of traditional remedies. This is consistent with previous studies in Kenya that reported the use of traditional remedies, such as tourniquets, black stones, and herbal medicines, before seeking formal healthcare [8, 21]. Although some plant species have been claimed to have antivenom properties, their efficacy and safety have not been verified [8]. Moreover, the use of traditional therapy may delay the administration of antivenom, which is the only effective treatment for snakebite envenoming [20]. Therefore, there is a need to educate the communities on the importance of timely access to health facilities and the dangers of using unproven methods. Additionally, there is a need to improve the availability and quality of antivenom which may encourage patients to seek care earlier [20].

We identified antivenom shortage and costs as major barriers to snakebite management. Antivenom is the only specific treatment for snakebite envenoming, but it is often scarce and unaffordable for many patients in sub-Saharan Africa [25, 26]. The average cost of antivenom per vial ranges from $18 to $200, and each patient may require up to 10 vials or more depending on the snake species and the severity of envenoming [27, 28]. However, most snakebite victims are poor rural dwellers who cannot afford such high costs. As a result, many patients resort to traditional healers who offer cheaper but ineffective and sometimes harmful remedies [29]. This situation calls for urgent interventions from the government and other stakeholders to ensure regular and adequate supply of quality-assured antivenom in the high-burden areas. Moreover, there is a need to subsidize the cost of antivenom and make it accessible and affordable for the patients who need it. Furthermore, there is a need to strengthen the health system capacity and the training of healthcare workers on snakebite management.

We found that healthcare workers had limited knowledge and skills to manage snakebites. This is a common challenge in the effective management of snakebite in Africa, where healthcare workers often lack formal training and clinical protocols for snakebite care [19]. Poor knowledge and skills can lead to incorrect or inappropriate treatment, such as using the wrong antivenom or dosage, applying harmful first aid measures, or failing to recognize the clinical features and complications of snakebite envenoming [19, 30]. Moreover, the training curriculum for clinicians in most African countries is inadequate and often does not include snakebite management. This highlights the need to identify the training needs and arrange for regular training of healthcare workers on snakebite management. Such training should cover topics such as local snake identification, snakebite first aid, antivenom administration and rational use, management of antivenom adverse reactions, and treatment of snakebite complications. Furthermore, there is a need to develop and disseminate evidence-based guidelines and protocols for snakebite management in different settings and levels of care.

The study revealed that poor referral systems and resources for snakebites are a significant challenge for many healthcare workers [31]. This challenge can lead to delays in treatment for snakebite victims, which can result in fatal or disabling outcomes such as tissue necrosis, hemorrhage, shock, or paralysis [32]. Therefore, it is essential to improve the referral system and provide adequate resources for snakebite management. Some possible strategies include: providing clear guidelines and protocols for snakebite referral and treatment [31], ensuring the availability and accessibility of effective antivenoms at different levels of health facilities, training healthcare workers on snakebite prevention and first aid, and equipping health facilities with transportation and communication means to facilitate timely referrals.

The study identified several opportunities to improve snakebite management, which can be grouped into two main categories: education and research. Education involves increasing awareness and knowledge about snakebite management among healthcare workers and the general public. Healthcare workers need to be trained on the treatment guidelines and the use of antivenom, as well as facilitate them to conduct outreaches and educate the community on snakebite prevention and first aid. This can enhance their confidence and competence in managing snakebite cases and reduce delays in seeking medical care. The general public also needs to be informed about the risks and signs of snakebite, the appropriate first aid measures, and the importance of accessing health facilities as soon as possible. Education can potentially reduce the morbidity and mortality associated with snakebite envenoming, as well as the social and economic burden on the affected individuals and communities [23, 33]. Research involves exploring better snakebite management practices and alternative treatments that can complement or replace antivenom, which is often scarce, expensive, or ineffective against some snake venoms. Research can also help to identify the epidemiology and ecology of snakebites, the clinical features and outcomes of different snake species, and the optimal dosing and administration of antivenom [23]. Research can contribute to improving the quality and availability of antivenom, as well as developing new interventions that can prevent or reverse the effects of snake venom. These opportunities for improvement are consistent with the World Health Organization’s guidelines and recommendations for managing snakebites.

### Strengths and limitations

The study provides valuable insights into the challenges and opportunities in snakebite management in Uganda, a country where snakebite is a neglected public health problem.

The study uses qualitative methods to explore the perspectives and experiences of healthcare workers who are directly involved in snakebite care, which can inform policy and practice recommendations. To further strengthen the credibility of our analysis, we adopted reflexivity and an iterative approach to data analysis, and refining our interpretations along the way.

However, it is important to acknowledge certain limitations when interpreting our findings. Our reliance on IDI interviews as the primary data gathering method may have restricted the breadth of information obtained, and the absence of corroborative data sources could have potentially impacted the data quality. Additionally, the study does not include the views and experiences of snakebite patients or their caregivers, which may provide additional insights into the barriers and facilitators of snakebite management. Furthermore, it is worth noting that our study was limited to one region of Uganda and therefore caution should be exercised when generalizing these findings to regions.

## Conclusions

Snakebite is a neglected and poorly managed public health problem in Uganda. This study explored the challenges and opportunities in snakebite management from the perspective of healthcare workers. The study found that healthcare workers face barriers such as lack of clinical guidelines, delayed health-seeking behavior, limited knowledge and skills, and antivenom shortage. The study also found opportunities such as increasing education and awareness, conducting research on alternative treatments, and securing funding for supplies. These opportunities align with the World Health Organization’s guidelines for managing snakebites. The study adds to the knowledge on snakebite management in Uganda and similar settings. The study also has policy implications for improving antivenom availability and quality, developing evidence-based clinical guidelines, and strengthening referral system and resources. The study also has practice implications for enhancing healthcare workers’ training and skills, conducting community education on snakebite prevention and first aid, and implementing integrated approaches to address snakebite patients’ healthcare needs.

## Supporting information

S1 File Themes and codes summary. (XLSX)

S1 Checklist COREQ (COnsolidated criteria for REporting Qualitative research) checklist. (DOCX)
